# The Long-Term Impact of Education on Diabetes for Older People: A Systematic Review

**DOI:** 10.5539/gjhs.v5n6p30

**Published:** 2013-07-28

**Authors:** Soontareeporn Thongsai, Malinee Youjaiyen

**Affiliations:** 1 Boromarajonani College of Nursing, Bangkok, Thailand

**Keywords:** systematic review of diabetes education, impact of diabetes education

## Abstract

**Background:**

Although enthusiasm is growing for diabetic education programs for older people, data regarding their effectiveness and their long-term impact on self-management were neglected.

**Purpose:**

To systematically review diabetes mellitus education that has long-term effects on the self-management of older diabetic people.

**Data Sources:**

The authors searched multiple sources dated through September 2012, including the Cochrane Library, MEDLINE, PsycINFO, Nursing and Allied Health databases, and the bibliographies of 50 previous reviews.

**Methods and Data Extraction:**

Electronic databases were searched for controlled studies in English, published from 1987 to 2012, assessing the effects of long-term education for older people. Reviewers extracted study data using a structured abstraction form. Aggregated information about the effects of long-term education programs on older people with diabetes was used for making adjustments in the review.

**Results:**

The pooled estimate of the long-term effects of education was a 0.5 percentage point reduction (95% confidence interval), modest but significant improvement. The evidence also supports that long-term education is beneficial for improving diabetic patient self-care management in terms of glycemic control.

## 1. Introduction

Chronic diseases are the main cause of death in the population today. In a recent study, it was argued that since the beginning of the 20^th^ century, the relative distribution of somatic illness in the population has changed and today chronic illnesses predominate. Diabetes mellitus is a chronic condition affecting the general population. Furthermore, there is some other evidence which supports this. Diabetes also potentially leads to disability and death for the people who have been diagnosed with it, as well as affects their family, who may have difficulties maintaining normal family life. This is because diabetes is a difficult condition to live with, and it is essential that the patient has knowledge about their illness in order to be able to control and cope with their situation, otherwise it might cause long-term complications. Hill (1987) suggested that diabetes mellitus and its long-term complications can be caused by several etiological processes, which has been an important step in the understanding of the disease. Also, some knowledge suggests that “diabetes is a complex chronic disease that affects every aspect of a person’s life and affects almost all organ systems. “The disease if not well managed, results may in increased morbidity and mortality at great economic and emotional costs” stated by Guthrie and Guthrie (1997). However, even today with advances in medical technology and the large amounts of research that have been done to establish supporting evidence to try addressing this problem, the number of people who have been diagnosed is still increasing every year. This will become a significant problem if we cannot control the progression of the disease.

Since the last century, there have been many studies in this area which have tried to classify and explore these problems. Even now, diabetes is still a big problem for patients who have diabetes and the staff who have tried to provide care for them. Especially for older people, who are a big portion of the diabetic population, this is very problematic. Once patients have been diagnosed, it may mean that they need to change their lifestyle or need to go to the hospital more often if they fail to control their disease. Not only is time spent undergoing treatment, but it may also lead to increased costs for treatment and finally end with organ damage because of complications of diabetes. The study by Leese (1991) reported that “the most important contributors to the direct costs of diabetes are those of treatment complications, such as eye and limb diseases, heart disease, neuropathy, nephropathy and indirect costs, such as the effects of time lost from work, early retirement and premature death”.

There are many studies that have been done in this area which have tried to explore the relationship between the patient’s education and glycemic control, which is the main factor that causes complications in diabetes. To date, there are some studies which have recognized that patient education will have an effect in the short-term for glycemic control, but not many studies have been done on the long-term impact. Heisler et al. (2005) stated that patient education facilitates self-management of diabetes and has evolved to become a cornerstone of quality-oriented diabetes care. In a systematic review on this subject, it will be interesting to find out the importance of education that may affect long-term outcomes for diabetes patients. Also, the results may be used as evidence to support professional health care staff in continuing education programs for diabetes patients.

## 2. Diabetes Mellitus

### 2.1 Definition

The World Health Organization (www.who.int/mediacetre, 2012) stated “diabetes mellitus is a chronic disease caused by inherited and/or acquired deficiencies in production of insulin by the pancreas, or by the ineffectiveness of the insulin produced. Such a deficiency results in an increased concentration of glucose in the blood, which can damage many of the body’s systems, in particular the blood vessels and nerves.” With type 2 diabetes and the progression of the disease, it is important to provide education for the patients so that they will be able to adapt to their situation.

### 2.2 Sugar Level or Blood Glucose

Definition: The World Health Organization, 2012 stated “impaired glucose tolerance (IGT) and impaired fasting glycemia (IFG) refer to levels of blood glucose concentration above the normal range, but below those which are diagnosed for diabetes mellitus”. Sugar level in the blood is very important in type 2 diabetes because, in this group, patients mostly have a problem with controlling their blood sugar levels. “In this type of diabetes, there is some insulin in the body but not enough to maintain good health, indicating that it usually occurs in middle and older aged groups” (Day, 1936).

### 2.3 Patient Education (Self-Management, Self-Care)

Definition: (www.joslin.org, 2012) stated “patient education, the basic concepts of the patient’s self-management, are the foundation for the patient education component of the disease management program.”

### 2.4 Elderly (Aged 65 and Over, Aged Group)

Definition: This definition will vary in different studies depending on the population being studied. This study will be based on the definition from The Public Health Agency of Canada which states that (http://www.phac-aspc.gc.ac, 2012) “seniors are the majority of participants 65 years and older.” In the last 20 years, there has been some evidence in this area which suggests that type 2 diabetes has affected the majority of the elderly group. There are increasing numbers of people who have been diagnosed with diabetes who are elderly. According to this statement, diabetes is a chronic disease which may affect almost all organ systems if not well managed, and the result will be increased morbidity and mortality, especially in the elderly group. These people may fail to control their disease, and they may have other health problems which make their management more difficult, such as obesity, coronary disease, and hypertension, which are related to the failure of health management. Hill (1987) suggests that diabetes mellitus and its long-term complications can be caused by several etiological processes, an important step in the understanding of the disease. Kesson and Knight (1994) state many elderly patients have little knowledge of how their body functions and often even less desire to gain such expertise”.

## 3. Theoretical Frameworks, National & International Context and Justification for Review

There are many studies of the incidence of diabetes which have supported the idea that patient education (self-management, self-care) is very important for improving the prognosis and life expectancy in patients with type 2 diabetes (non-insulin dependent) (Steed et al., 2005) argued that self-management has been described as the cornerstone of care for diabetes, especially in elderly people who have a high incidence of the disease.

## 4. Search Methodology

The comprehensive search methodology was developed based on the systematic review guidelines of the National Health Services Centre for Review and Dissemination at University of York (http://www.york.ac.uk, 2012) and the search strategy developed by Robin Snowball of the Cairns Library and the Canadian Institutes of Health Research was used. Also, the framework in clinical issue search strategies produced by Cochrane Collaboration (Cochrane Database of Systematic Review) (http://www.exampla.ors, 2012) was used. The selection of the articles was classified to ensure the inclusion of as many relevant published studies as possible for the first screening. Articles which were not published in the English language will be selected as relevant data and may be included in the review later.

## 5. Sources

The sources used in this review included: CINAHL (Cumulative Index to Nursing and Allied Health Literature), a database which covers health, social work and education articles published in journals.

HSWE Database: this database covers articles published in journals taken at Coach Lane Library since 1997 and it also links to government and other publications available on the internet.

ProQuest Nursing Journals database: this database is a searchable database of 250 journals in nursing and allied health.

All of the articles were published between 1982 and May 2012, because the sources for this study were selected from CINAHL, HSWE, and the ProQuest Nursing Journals database, and the earliest of the articles included in these databases started from 1982 and continued through 2012. The web link from the Northumbria University at Newcastle upon Tyne (www.gateway.uk.ovid, 2012) stated Cumulative Index to Nursing & Allied Health provides authoritative coverage of the literature related to nursing and allied health from 1982 to July 2012. All publications were in the English language. The studies were original articles reporting the results of randomized controlled trials of the effectiveness of education (self-care, self-management) for glycemic control in type 2 diabetes patients. For the qualitative study, the focus was on case management, which is related to the risk and the benefits of glycemic control. Relevant data on the study design, long-term impact of education, intensive education, achieving glycemic control, self-monitoring of glycemic levels, methodological quality, and inter-external validity will be explored. Other relevant databases searched, such as HMIC (Health Management, Health building) and Health, lifestyle will have an emphasis on education in terms of self-care and lifestyle behaviors. These two databases mainly provide the subject of health service policy, healthcare management and hospital administration with an emphasis on the British National Health Service. Because of this, these two databases will be beneficial to use to find out about the health lifestyle which is associated with this review.

## 6. Search Criteria

Guidelines were used which described the seven steps of the review process for finding information from research papers and documents. These consisted of a three-step screening process, and it was used as described below for the study:

Step 1: Classifying (locate all of the documents by using search strategies to classify)
Step 2: Screening for the selection of abstracts, Documents and full texts
Step 3: Classifying papers by relevance for the systematic review and randomized controlled trial.

## 7. Objectives

1). To identify systematic reviews, the randomized control trials which have the efficiency of education in diabetes patient in elderly group
2). To review and appraise the findings of the study

## 8. Search Strategies Format

## 9. Study Question

Is education effective for glycemic control in type 2 diabetes patients over 65 years of age in terms of:

- Long-term educational impact?
- Self-care, self-management?

## 10. Selection of Studies (Key Criteria Identified)

There are two designs which were eligible for the review: randomized control trials (RCT) and systematic reviews. The studies have to be published in English to be eligible for the full review. This is because of time constraints and also because the results will be only provided as an evidence base to support health care professionals in deciding if long-term education for diabetes patients is possible. However, if there are some papers published in other languages containing English abstracts which contain very relevant issues, it may be included in the review later on. Also, the selection of this study was mentioned above.

## 11. Type of Participant

### 11.1 Education (Self-care, Self-management, Patient Education)

The study selection criterion for the type of studies includes educational terms such as self-care, self-management and patient education. The aim is to identify the effectiveness of education that affects the control of glycemic levels.

### 11.2 Elderly Group (Aged 65 and Over, Aged Group)

This study selection criterion places emphasis on people 65 years and older which have been diagnosed and defined by the ninth revision of the International Classification of Disease (ICD)-9. The aim is to verify the criteria and avoid the different factors that could be involved.

### 11.3 Glucose and/or Glycemic Control

This study selection criterion focuses on patients with type 2 diabetes who have a problem with glycemic control. The aim is to find out the relationship between two factors (self-management, self-care, patient education and glycemic control).

However, because there may be many studies which have been done in this area, it is impossible to include all of that research within the time constraints of this study as noted previously. Glaziou and Coldditz (2001) have stated that “finding all relevant studies is almost impossible.” In attempting to gather as many relevant studies as possible in the time that was available, the following methods were used. I carried out an electronic search of the databases by using three step search strategies in order to classify all relevant papers. This was done because, with the three step search process, a consistent result will be produced which avoids other factors that may cause variation such as researcher bias. The search strategy was also developed under consultation of the librarians at Northumbria University of Newcastle upon Tyne.

Relevant web sites were explored for useful information, such as WHO (World Health Organization), Department of Health, and the Clinical Diabetes, which have the most relevant and fundamental information and receive regular updates.

## 12. Searching of Electronic Databases

**Figure 1:**
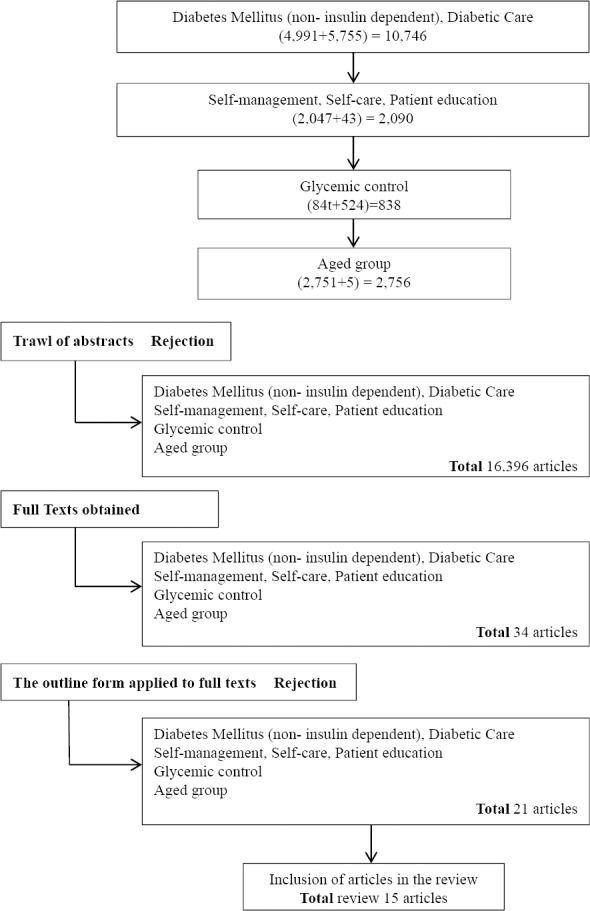
The flow diagram of the study selection

Figure 1 shows the flow diagram of the study selection.

Cinah, Pro Quest Nursing Journal, HSWE (Health, Social work and education articles a published in journals taken at Coach Lane Library science 1997)

Identification of abstracts: All of documents in Total were count from Cinah and Pro Quest Nursing Journal in sequence.

## 13. Validity and Reliability

The strength of the studies will be an important feature, because if any studies have an effect on the assessment and process of search strategies then the result will be shown in the validity and reliability of the studies. On the other hand, if any studies show weakness in the process, then the quality of the studies will be inconsistent. To avoid this error, the validity and reliability of the studies needs to be looked at carefully. Due to this, the review stage is intended to find the best and strongest available evidence that has been shown.

However, there are some variations and differences in study quality that may affect the final results. In order to get the best quality evidence, I needed to develop a method for the standardized process of quality appraisal.

## 14. Data Extraction

All studies that met the relevant criteria were retained for the data extraction stage of the review process. A data extraction form was developed based on a guideline developer’s handbook for systematic review, randomized controlled trial and case control studies (http://www.sign.ac.uk, 2012). The data extraction will include information on the study design, participants, intervention approaches, self-management/self-care/patient education definitions, study outcome, and issues with implementation. However, section three (the pilot stage by second observers) of each guideline needed to be adapted by piloting with secondary observers who will be involved in this stage as consultants for the study, to be able to avoid primary author bias and for making decisions about which criteria need to be added in order to extract more relevant information. Unfortunately, this systematic review study was limited by time, and this part will be based solely on adapting my understanding.

**Table 1:** Summary of the study

Study	Intervention Strain of Self-management (education)	Country of origin	General inclusion/exclusion criteria	QA score
Davis, E. “A quality& improvement Project in Diabetes education	T	United Kingdom	Meet criteria	22
Weinman, J. hospitalization Fox, C- (2)3 “Intensive emotion for lifestyle change in diabetes”	T	United State of America	Meet criteria	22
Fonseca, Y. “Long-term effects of self- management education for Patient type 2 diabetes taking maximal oral hypoglycemia therapy”	T	United State of America	Meet criteria	20
Heisler, M. “The relationship between knowledge of recent values and diabetes care understanding and self- management	T	United State of America	Meet criteria	24
Lzquierdo, R. “A comparison of diabetes education administered through telemedicine versus in person”	T	United Kingdom	Meet criteria	22
Norris,S. “Effectiveness of self-management training in type 2 diabetes”	T	United State of America	Meet criteria	22
Nod, S. “Effectiveness of self- management training in type 2 diabetes : systematic review”	T	United State of America	Meet criteria	20
Norris, S. “Self- management education for adult with type 2 diabetes Sarkisian, C.	T	United State of America	Meet criteria	24
systematic review of diabetes self-Care Interventions for Older, African American, or Latino”	T	America Africa	Meet criteria	22
Sone, H. “The long-term effects of self-management education for Patients with type} diabetes on glycemic control”	T	Japan	Meet criteria	20
Down, SH. and Black, N. “The feasibility of creating a checklist for the assessment of the methodological quality studies of health care interventions”, Journal of Epidemiology & Community Health	T	United State of America	Meet criteria	20
Ockleford, E., Shaw, R. L., Willars, J., & xon-Woods, M. 2008, “Education and self-management for people newly diagnosed with type 2 diabetes, *Chronic Illness,*	T	Australia	Meet criteria	22
Steed, L. & Lankester,J. & Barnard, M. et al “Evaluation of the UCL Diabetes Self-Management Program (UCL-DSMP): a randomized controlled trial”	T	United State America	Meet criteria	22
Richter, B. & Berger, M. “RCT remain fundamental to clinical decision making in Epe2 diabetes mellitus: a comment to the debate on RCT”, Diabetologia	T	Japan	Meet criteria	20
Patel, V. L., Kushniruk, A. W., Yang, S., & Yale, J. F, “Impact of a Computer-based Patient Record System”, Journal of the American Medical Informatics Association	T	New Zealand	Meet criteria	20

## 15. Data Synthesis: Presenting the Findings and Results

The final decision on how to present the results was made at the last stage of data summarization of the included papers. The decision was based on the nature of the results of the different studies.

## 16. Results: New Understanding

After reviewing the entire documents that met the criteria, many gaps are clearly evident and, additionally, there are some new understandings which have emerged. A total of 43 published articles were identified. These studies are associated with long-term education, effectiveness in self-management, patient education, glycemic control outcome studies, and study quality. Review of this literature was based on the effectiveness of self-management and glycemic control in patients with type 2 diabetes described in the paper. Educational programs with follow-ups ranging from six months to one and a half years were selected to identify long-term impact. Improved glycemic control was associated with weight loss in some studies. Also, increased time spent in physical activity was found to have improved glycemic control in only one study, although another study reported no change in the final result if the educational programs were followed up longer than one and a half years. Finally, to be able to find out that an effective self-management program or better (patient education, self-care) was related to the improvement of glycemic control with type 2 diabetes patients. Even in long-term education, there were no significant differences in terms of the therapeutic content. Not only this, but also there were some new findings located that suggest that in the last 10 years, most concepts related to health care or education programs mostly focused on the professional perspective which simply concentrated on ways to tell the patient to do whatever that the health care team had knowledge of but was not concerned about patient satisfaction and all other factors that may be associated with diabetes. This results in failing to develop an education program, which is paramount for the improvement and prevention of chronic diseases such as diabetes mellitus. Fox and Kilvert (2003) argued that traditional education for diabetes treats the patient as a receptacle for knowledge or a pot to be filled with information by doctors, nurses, and dieticians. Reflecting on this point, this failure in health education should be decreased because now all of the health care teams are more concerned with the new concept that considers the importance of the individual needs and sets up the goal as a collaboration to achieve success in metabolic control, as well as to prevent acute and chronic complications. The American Association of Diabetes Educators (http://www.aadenet.ore, 2012) stated “training in self-management is integral to the treatment of diabetes. Treatment must be individualized and must address medical, psychosocial and lifestyle issues”. Furthermore, with the diabetes education program, there is some evidence which supports the idea that diabetes self-management training programs had achieved an improvement in glycemic control, especially in adult groups. Diabetes care (http://www.care.diabetesjournals.org, 2012) argued “diabetes self-management training, the process of teaching individuals to manage their diabetes, has been considered an important part of clinical management since the 1930s,” but unfortunately this systematic review focused on the elderly group and included some of the evidence from studies which had a target group of adults in general, without separating the data from older people. An example is the studies of Fonseca in 2004 with an adult group, but the findings of the studies included patients from 39-75 years of age. This study was therefore included after using search strategies because it included the elderly, which is the target group for this review.

Short-term education is an effective course for the improvement of controlling glycemic levels in patients with type 2 diabetes. (Norris et al., 2012) stated that positive effects of self-management training on knowledge, frequency and accuracy of self-monitoring of blood glucose, self-reported dietary habits, and glycemic control were demonstrated in studies with short follow-ups (less than 6 months of monitoring). In the last 10 years, short-term intervention has been recognized as effective for patients with type 2 diabetes, but the long-term impact is still not clear. However, after reviewing all of the articles that had been located and met the criteria, there was some evidence from longer follow-ups which continued monitoring for diabetes education that showed that it was effective in the long-term. Finally, it was found that a diabetes education and self-management program can be combined with new technology with results as successful for the glycemic control patient group as the original diabetes education group. There are some studies that have supported the idea that patient education (self-management, self-care) diabetes education can be provided as an effective program through new technology such as telemedicine. This is an advancement of the education program that, in the future, will be part of all training for improvement of the quality of life for patients, particularly those patients with type 2 diabetes, in education programs.

## 17. Conclusion

Finally it was found that a diabetes education, self-management program can be combined with new technology and results were as successful for the glycemic control patient group as for original diabetes education. There are some studies that have supported the idea that patient education (self-management, self-care) diabetes education can be provided as an effective program through new technology such as telemedicine. Lzquierdo et al. (2003, p.1002) Stated “Diabetes education via telemedicine and in person was equally effective in improving glycemic control, and both methods were well accepted by patients”. This is an advance of the education program that in the future will be located with all of education program training for improvement of the quality of life for patients particularly, for diabetes patient with type 2 diabetes.

## 18. Limitations of the Systematic Review

The advantage of the systematic review is that it might be an effective tool to help healthcare teams and staff to be able to integrate their knowledge and new information in a beneficial way. Egger (2001) argued that there are approximately 17,000 new biomedical books published every year, along with 30,000 biomedical journals with an annual increase of year. This makes it very difficult for health care professionals to keep on top of the most recent advances and research in their field, as they would need to read, on average, 17 original articles each day. To make this task slightly more manageable, healthcare providers and other decision-makers now have, among their information resources, a form of clinical report called the systematic review. Also, the results from the systematic review will be applied to clinical practice and used as a guideline to adapt and improve their skills and knowledge of staff. However, all of these benefits will be present only whenever there are many studies available in that area. Accordingly, because there are not many studies that have been carried out in this topic, the systematic review approach is limited. The review was of a specific area especially relating to older people, and there were a small number of relevant studies to review. In addition, there are geographical differences, as some countries are addressing this need and some do not recognize its importance in this situation. Steed et al. (2005) stated that self-management has been described as the cornerstone of care for diabetes and many self-management studies are limited by poor methodology and poor descriptions of the intervention. Reflecting on this point, this may be another reason that there is a small amount of studies in this area of review. Even in the United Kingdom, which has an advanced professional healthcare system, there are a small amount of articles. Fox and Kilver (2003) argued “the United Kingdom lags far behind; although the National Service Framework for diabetes has recognized that the provision of information, education, and psychological support that facilitates self-management is the cornerstone of diabetes care and has set primary care groups the target of providing empowering education by March 2006.” However, there are quite a few studies and systematic reviews recommending the effectiveness of self-management, self-care or patient education in improving glycemic control and which might delay the use of insulin as a treatment for patients with type 2 diabetes. Because this study was done with older adults and recommended short-term education, this article would be rejected at the end of the search strategies process. Not many studies have been done in this area, but some of articles are only available as an abstract, which generally have insufficient information to assess the validity of the study and criteria, and this factor directly affected the review of the studies. The other factor which decreased the number of articles in this field was the limitations of the language the studies were published in, because part of the criteria for review required the articles to be published in the English language. Unfortunately, quite a few of the documents presented abstracts in the English language but later it was found that the articles were published in another language such as French or Chinese, as detailed in this article Sarkisian: A systematic review of Diabetes Self-Care Interventions for Older, African, or Latino Adults by Sarkisian and Brown (2003). Lastly, a limitation of this systematic review was the process and time of the systematic review. It is recommended that more than three people be involved in the review process to diminish bias in the studies. Because of the time constraints, this was not possible in this review. Also, sometimes it may be necessary to have a pilot stage to develop a form that may be used for classifying articles. For this systematic review, only one person carried out this process under supervision and the process in its entirety was carried out with limited time, which may have affected the result of this systematic review at some point.
